# A novel method for extraction of a proteinous coagulant from *Plantago ovata* seeds for water treatment purposes

**DOI:** 10.1016/j.mex.2015.05.006

**Published:** 2015-05-26

**Authors:** Bahman Ramavandi, Seyedenayat Hashemi, Raheleh Kafaei

**Affiliations:** Environmental Health Engineering Department, Faculty of Health, Bushehr University of Medical Sciences, Bushehr 7518759577, Iran

**Keywords:** Water turbidity coagulant extracted from *Plantago ovata* seeds by using an FeCl_3_-induced crude extract, *Plantago ovata* seeds, Protein, Extraction, Coagulant

## Abstract

Several chemicals have been applied in the process of coagulant extraction from herbal seeds, and the best extraction has been obtained in the presence of KCl or NaNO_3_[1], [2], [3], and NaCl [4]. However, the main challenge posed to these methods of coagulant extraction is their relatively low efficiency for water treatment purposes and the formation of dissolved organic matter during the treatment process. In these methods the salts, which have a one-valance metal (Na^+^ and K^+^), are deposited in the internal structure and the pore of the coagulant, and may be useful for the coagulation/flocculation process. In this research, we found that modified methods produced more dense protein. Therefore, the modified procedure was better than the older one for removal of turbidity and harness from the contaminated water. Here we describe a method where:

• According to the Hardy–Schulze rule, we applied the Fe^3+^ ions instead of Na^+^ and K^+^ for the extraction of protein from *Plantago ovata* seeds.
• The method was narrowed to extract protein by ethanol (defatting) and ammonium acetate and CM-Sepharose (protein extraction).
• Two consecutive elutriations of crude extract was directly performed using 0.025-M FeCl_3_ and 0.05-M FeCl_3_ according to the basis of the ion-exchange processes.

## Graphical abstract

**Figure:**
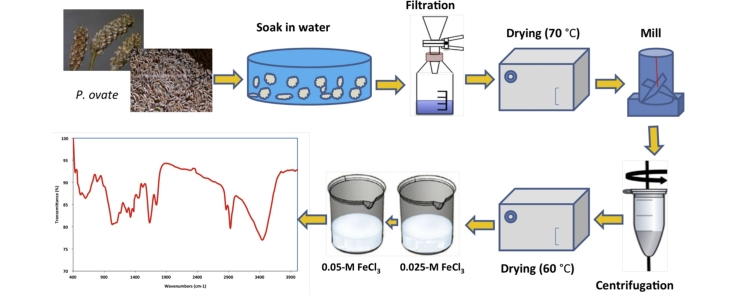

## Method details

1. Dry *Plantago ovata* seeds
2. Ammonium acetate
3. Sodium hydroxide
4. Hydrochloric acid
5. Ferric chloride
6. Calcium chloride
7. Ethanol (98%)
8. CM-Sepharose
9. Distilled water
10. Membrane filter (0.42 μm)

## Extraction and purification processes of coagulant from *Plantago ovata* seeds

Extraction of the crude coagulant from *P. ovata* seeds was done as follows:

○ The seeds were soaked in distilled water for 1 day.
○ The obtained gelatinous material was passed through 0.42 μm membrane filters and dried at 70 °C in an oven and then milled in a domestic blender (VARING).
○ The obtained powder was defatted by mixing it with ethanol 98% using a magnetic stirrer for 60 min. Then, the supernatant was separated by centrifugation (3500 rpm, 40 min), and the settled powder was dried overnight in an oven (at 60 °C).
○ The crude coagulant was extracted from the oil-free powder using 10-mM ammonium acetate in 5% w/w. The mixture was stirred for 50 min using a magnetic stirrer, and the supernatant, namely the crude extract, was separated by centrifugation (3500 rpm, 40 min).

The purification of the coagulant protein from the crude extract was carried out as follows:

○ CM-Sepharose ion exchange was equilibrated using a 10-mM ammonium acetate solution.
○ Equilibrated CM-Sepharose ion exchange was added to the crude extract in 10% (v/v) proportion and mixed using a blade stirrer for 50 min.
○ Finally, the absorbed coagulant protein was eluted by different concentrations of an FeCl_3_ solution.

The purification procedure was performed directly with 0.025-M FeCl_3_ first and then with 0.05-M FeCl_3_ elution according to the basis of the ion-exchange processes [4], [5]. In the first elution, proteins that presumably did not lead to a higher coagulation performance (efficiency less than 45%) and simply added dissolved organic carbon (DOC) (about 3.5 mg/L) in the treated water were removed. Consequently, the second elution produced a more purified coagulant as it contained only the active coagulant proteins. The elution stages were repeated three times to recover as much coagulant as possible. Further, after the both purification of extraction by FeCl_3_, the resultant coagulant was rinsed many times with double distilled water until Fe ions not detectable in rinsed water. The coagulant obtained in the second elution was removed 99% of water turbidity and added only 0.8 mg/L of DOC to the treated water.

## Additional information

**Fig. 1:**
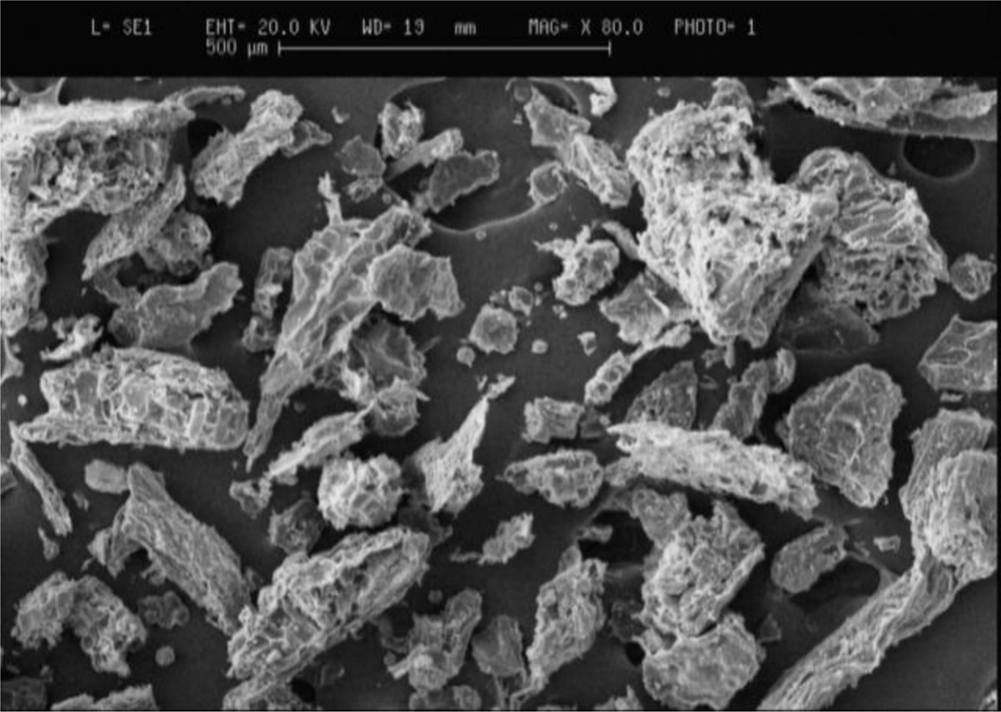
The SEM image of sludge after water turbidity removal by the coagulant extracted from *Plantago ovata* seeds.

**Fig. 2:**
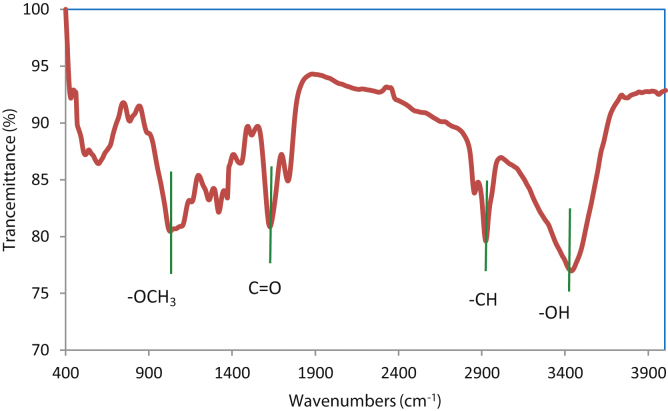
FTIR spectra of extracted protein from *Plantago ovata* seeds.

The extracted coagulant was applied for removing of turbidity from water. Here, the scanning electron microscope (SEM) micrographs of the settled floc (sludge) during water treatment by the coagulant and the Fourier transform infrared spectroscopy (FTIR) of the extracted coagulant are discussed. From the SEM micrographs (Fig. 1), it is clear that the sludge after the water treatment with extracted protein are hard and sturdy. The honeycomb-like structure, with small colloidal particles entrapped in it, might indicate the contribution of the sweep flocculation mechanism for the removal of colloidal particles in the water mixture. The FTIR spectra of extracted protein presented in Fig. 2 indicates several main peaks at 3410, 2920, 1604, and 1000 cm^−1^ on the spectra which corresponds to —OH groups, —CH groups, C=O bands, and —OCH_3_ groups [6], [7]. These active groups on the surface of extracted proteinous coagulant may be involved in turbidity removal from waters. The chemical composition of the sludge and fresh extracted coagulant is given in Table 1. Among various elements, carbon was found to be dominant in the sludge. Regarding to polysaccharide [C*_n_*(H_2_O)*_m_*], fatty acids [CH_3_—(CH_2_)*_n_*—COOH], and protein general formula [RCH(NH_2_)COOH], the obtained coagulant could be neither polysaccharide nor fatty acid as it contained N. Also, regarding to mentioned formula, the polysaccharides contain high amount of oxygen rather than proteins and fatty acids, so the coagulant structure could not be made from polysaccharide. Further, some amino acids such as *met-* and *cys-*amino acids contain S, thus presence of S in the structure of coagulant might be another reason for proteinous of the extracted coagulant. However, low values of P may be due to phospholipids acids in the extracted coagulant. The proteins namely metallo-protein contain metals such as Fe, however we modified the crude extract with FeCl_3_. Na and other such metal is naturally presented in plant seeds. Thus, it can be concluded that the extracted coagulant is majority protein and minority phospholipids acids.

**Table 1:** The EDAX analysis of the crude extract, fresh extracted protein, and sludge during water treatment.

Element (wt%)	Value		
	Crude extract	Fresh extracted protein	Sludge
C	22.10	24.15	58.07
N	0.72	0.82	1.91
O	0.61	0.63	0.761
S	2.22	2.98	3.85
Al	0.16	0.170	0.132
P	0.77	0.52	0.61
Fe	0.64	4.77	4.01
Na	3.01	5.11	6.22
Cl	0.22	2.132	5.80

**Fig. 3:**
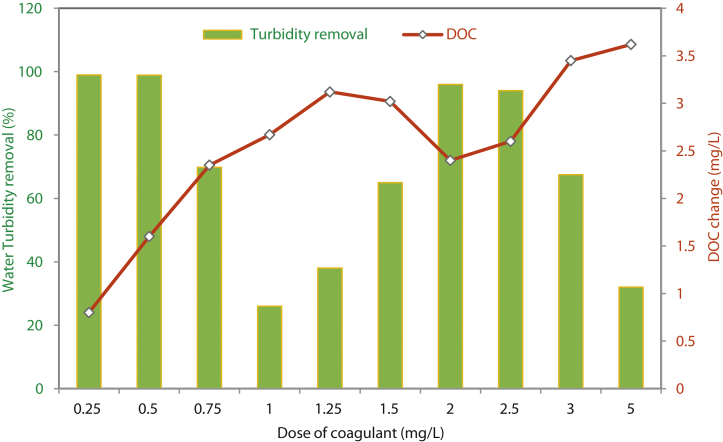
Effects of the coagulant dose on water turbidity removal and DOC change in treated water.

The coagulation process is usually a surface phenomenon; therefore, the performance a coagulant can be significantly influenced by the surface charge due to the mass of the coagulant. Thus, the optimization of the coagulant dose and the best-required mass of the coagulant for the scale-up and design of large-scale equipment is economically necessary. Hence, the effect of coagulant dose on the water turbidity removal was considered at the water pH of 7; the obtained results are plotted in Fig. 3 from the perspective of the turbidity removal efficiency and change of the DOC of the treated water. As can be inferred from Fig. 3, for the two lowest coagulant dosages, *i.e.*, 0.25–0.5 mg/L, the final turbidity was low, less than 5 NTU, the maximum admissible concentration according to European directive [8] for drinking water. Further, the results of the effect of coagulant dose on the turbidity removal exhibited the various trends as follow: First, the removal efficiency of water turbidity decreased from 99% to 26% as the coagulant dose increased from 0.25 to 1 mg/L. Then, the turbidity removal efficiency increased with an increase in the coagulant dose from 1 to 2 mg/L. Finally, the turbidity removal efficiency decreased when the coagulant dose was more than 2 mg/L (Fig. 3). This phenomenon could be explained by a couple of reasons. On one hand, at a lower coagulant concentration, its long chain adsorbed on the surface of one colloid particle was adsorbed onto the surfaces of the others, and therefore two or more particles aggregated by bridging flocculation. However, when the coagulant dose was increased to a certain value (here, 1 mg/L), the adsorbed aggregation completely covered the particle surface and prevented the particles from flocculating. On the other hand, under experimental conditions and pH = 7 < pH of zero point charge of the coagulant (that equal to 7.9), the coagulant was positively charged as the functional groups bonded with hydrogen ions. Therefore, when the coagulant dose was increased to 2 mg/L the positively charged coagulant adsorbed on the surface of the negatively charged colloid particles by charge neutralization mechanism and resulting a better coagulation effect.

The use of natural coagulants might increase DOC load in water, which may be resulted the in increasing of microbial activity of the treated water. Further, DOC might consume additional chlorine in the water treatment plant and act as a precursor of toxic byproducts during the disinfection process. Thus, the DOC changes during the tests were monitored. As can be seen from Fig. 3, the DOC change during the turbidity removal in the case of all the tested coagulant doses was less than 3.6 mg/L. However, the DOC change was only 0.8 mg/L for the optimum coagulant dose of 0.25 mg/L, implying that the background DOC 19.9% was increased. The DOC induced to treated water as compared to other coagulants [1], [2], [3], [4] used for turbidity removal was very low.
